# Social Motility of African Trypanosomes Is a Property of a Distinct Life-Cycle Stage That Occurs Early in Tsetse Fly Transmission

**DOI:** 10.1371/journal.ppat.1004493

**Published:** 2014-10-30

**Authors:** Simon Imhof, Sebastian Knüsel, Kapila Gunasekera, Xuan Lan Vu, Isabel Roditi

**Affiliations:** 1 Institute of Cell Biology, University of Bern, Bern, Switzerland; 2 Graduate School of Cellular and Biomedical Sciences, University of Bern, Bern, Switzerland; University of California, Los Angeles, United States of America

## Abstract

The protozoan pathogen *Trypanosoma brucei* is transmitted between mammals by tsetse flies. The first compartment colonised by trypanosomes after a blood meal is the fly midgut lumen. Trypanosomes present in the lumen—designated as early procyclic forms—express the stage-specific surface glycoproteins EP and GPEET procyclin. When the trypanosomes establish a mature infection and colonise the ectoperitrophic space, GPEET is down-regulated, and EP becomes the major surface protein of late procyclic forms. A few years ago, it was discovered that procyclic form trypanosomes exhibit social motility (SoMo) when inoculated on a semi-solid surface. We demonstrate that SoMo is a feature of early procyclic forms, and that late procyclic forms are invariably SoMo-negative. In addition, we show that, apart from GPEET, other markers are differentially expressed in these two life-cycle stages, both in culture and in tsetse flies, indicating that they have different biological properties and should be considered distinct stages of the life cycle. Differentially expressed genes include two closely related adenylate cyclases, both hexokinases and calflagins. These findings link the phenomenon of SoMo *in vitro* to the parasite forms found during the first 4–7 days of a midgut infection. We postulate that ordered group movement on plates reflects the migration of parasites from the midgut lumen into the ectoperitrophic space within the tsetse fly. Moreover, the process can be uncoupled from colonisation of the salivary glands. Although they are the major surface proteins of procyclic forms, EP and GPEET are not essential for SoMo, nor, as shown previously, are they required for near normal colonisation of the fly midgut.

## Author Summary

African trypanosomes, single-celled parasites that cause human sleeping sickness and Nagana in animals, are transmitted by tsetse flies. Bloodstream form trypanosomes ingested by tsetse differentiate into procyclic forms in the midgut lumen of the insect. Successful transmission to a new mammalian host requires at least two migrations within the fly: one from the midgut lumen to the ectoperitrophic space, and a subsequent migration from the ectoperitrophic space to the salivary glands. Procyclic forms can exhibit social motility, a form of coordinated movement, on semi-solid surfaces. While social motility in bacteria is linked to virulence, the biological significance for trypanosomes is unknown. We demonstrate that social motility is a property of early procyclic forms, which are equivalent to the forms present during the first week of fly infection. In contrast, late procyclic forms characteristic for established infections are deficient for social motility. Our findings link social motility to a biological process, confirm that early and late procyclic forms are distinct life-cycle stages and imply that genes essential for social motility will be of key importance in fly transmission. We suggest that using the social motility assay as a surrogate for fly experiments should enable many more laboratories to examine this aspect of parasite transmission.

## Introduction

Various sub-species of the protozoan parasite *Trypanosoma brucei* cause sleeping sickness in humans and Nagana in domestic animals. Irrespective of their mammalian host range, all these parasites are dependent on tsetse flies for their transmission. Two features enable trypanosomes to establish chronic infections in the mammalian host - their ability to evade the immune response by periodic switching of their variant surface glycoprotein (VSG) coat (reviewed in [1]) and a quorum-sensing mechanism that drives the differentiation of proliferating slender bloodstream forms to non-dividing stumpy forms, thus limiting the parasitaemia [2], [3]. Stumpy-inducing factor (SIF) is a small molecule (<500 Da) produced by the slender forms; its chemical identity is not known. Stumpy forms are pre-adapted for further differentiation and, following ingestion by the tsetse fly, differentiate into early procyclic forms in the lumen of the insect midgut [4]. In addition to changes in morphology and metabolism, differentiation involves the replacement of the VSG coat by two insect-specific coat proteins, GPEET and EP procyclin. At the beginning of tsetse infection procyclic forms can have two fates: they can be eliminated by the fly or they can migrate across/around the peritrophic matrix and colonise the ectoperitrophic space [5]. Once the infection is established, it is characterised by late procyclic forms that express high levels of EP, but are negative for GPEET. GPEET is not required for migration to the ectoperitrophic space, since deletion mutants can establish infections at normal rates [6].

Early and late procyclic forms can be maintained in axenic culture. Addition of glycerol to the culture medium prolongs the expression of GPEET; once glycerol is removed, the cells undergo a transient growth arrest and GPEET is repressed within a few days [4], [7]. Different trypanosome stocks vary in the relative amounts of GPEET or EP that they express in culture [8]. In contrast to what is observed in tsetse, GPEET-negative cells can revert to being GPEET-positive in culture, for example in response to glucose depletion [9] or by an unknown mechanism that is independent of glycerol [10]. To complete the cycle in the fly, parasites must migrate from the midgut, via the proventriculus, to the salivary glands. This migration constitutes a major bottleneck in the life cycle [11]. Once they reach the salivary glands trypanosomes attach to the epithelia and proliferate as epimastigote forms, finally giving rise to infectious metacyclic forms that can infect a new mammalian host.

Unicellular organisms can function as multicellular communities that exchange signals with each other and move in a coordinated manner. This is particularly well described for bacteria, which can form biofilms, communicate by quorum sensing and exhibit adventurous or social motility (SoMo) [12]–[15]. These types of concerted behaviour have implications for virulence and present potential targets for new classes of antimicrobial drugs. In contrast to what is known about social behaviour in prokaryotes, there is considerably less information on social interactions between unicellular eukaryotes. While several species of fungi are capable of forming biofilms [16], studies of swarming motility have focused almost exclusively on the free-living social amoeba *Dicytostelium discoideum* [17], [18]. In general, unicellular parasites tend to be studied as isolated entities or as organisms that need to perceive and interact with their hosts, with relatively little attention being paid to how they communicate with each other [19].

Procyclic forms of *T. b. brucei* exhibit SoMo when plated on a semi-solid surface, in a manner reminiscent of swarming bacteria [20]. Parasites first grow at the site of inoculation, and then form radial protrusions or “fingers” that extend outwards. Independent communities are able to sense each other and reorganise group movement to prevent contact. Migration on plates is abolished if the trypanosomes have a dysfunctional flagellum [20] or other motility defects [21]. It has been hypothesised that the social motility observed on plates might reflect one of the migration steps within the fly vector, either from the midgut lumen to the ectoperitrophic space, or from the ectoperitophic space to the salivary glands [20]. By using a series of mutants that had previously been characterised in tsetse, we show that SoMo is unrelated to the parasites' ability to establish salivary gland infections. Instead, it is a property of the early procyclic form, which is found in the first few days after transmission of bloodstream forms to the tsetse fly. We have also identified several new markers in addition to GPEET that are differentially expressed in early and late procyclic culture forms, and verified their differential expression in tsetse flies. Taken together, this confirms that early and late procyclic forms are distinct life-cycle stages with specific expression profiles and characteristics and links SoMo to an early event in the colonisation of the tsetse midgut.

## Results

### The time-point of migration correlates with the density of the inoculum

**Figure 1:**
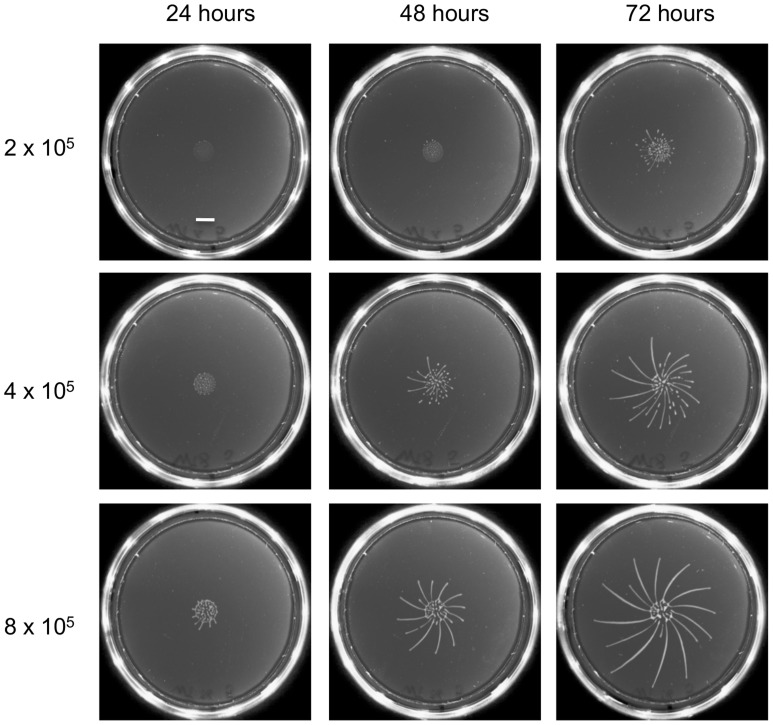
The time-point of migration is density dependent. Different numbers of early procyclic forms of AnTat 1.1 were resuspended in 5 µl and pipetted onto 0.4% agarose plates. Photographs of the plates were taken every 24 h. The scale bar is 1 cm.

**Figure 2:**
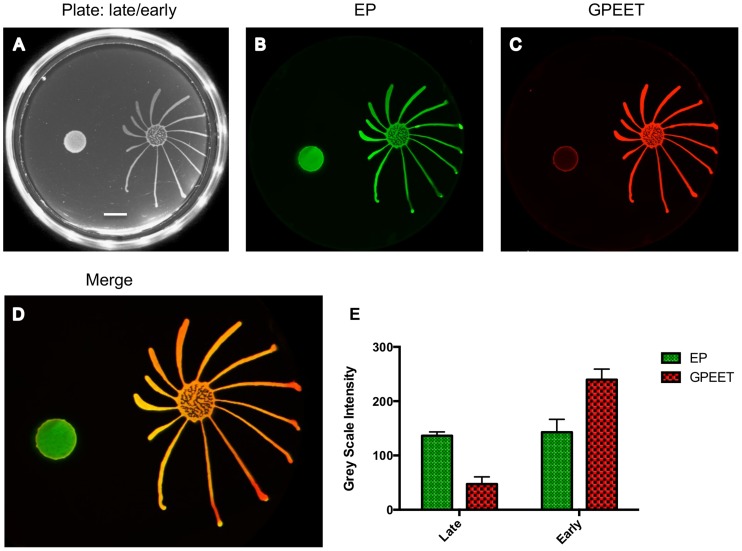
Only GPEET-positive cells exhibit SoMo. 2×10^5^ procyclic forms growing in medium with or without 20 mM glycerol were inoculated on an agarose plate containing 20 mM glycerol. (A) Photograph of the community 5 days post plating. Scale bar is 1 cm. (B–D) A community lift incubated with α-EP and α-GPEET antibodies. (E) Quantification of signal intensities. The intensity of EP is comparable between the early and the late procyclic communities. GPEET is predominantly expressed in the early, migrating colony. The error bar shows the mean standard deviation of seven individual areas.

As a first step we optimised the plating protocol for the fly-transmissible strain AnTat 1.1. The main differences from the previously published protocol [20] are that we used SDM79 rather than SM as the medium and cells were not preincubated with ethanol before plating. In addition, low melting temperature agarose was replaced by normal agarose, rendering the plates more robust. While establishing the SoMo assay we observed that the time-point when radial protrusions formed differed between experiments. To test if the cell density influenced the assay, different numbers of cells were pipetted onto the plates (Figure 1). When 8×10^5^ cells were plated in a volume of 5 µl, fingers were already visible after 24 hours. Cells plated at a density of 4×10^5^ or 2×10^5^ cells in 5 µl showed SoMo after 48 or 72 hours, respectively. It was reported previously that the doubling time of trypanosomes on plates is 24 h [20]. This suggests that the cells reach a threshold number of approximately 1.6×10^6^ before migration starts. We observed that when communities were plated on their own, the radial projections always grew in a clockwise direction (Figure 1, 72 h). This directionality was overridden, however, when cells sensed and avoided neighbouring communities (Figure 2).

### Social motility is a property of early procyclic forms

When we tested a variety of mutants, the high frequency of clones that were SoMo-negative, coupled with the observation that some addback mutants gave inconsistent results, made us suspect that a factor unrelated to the genotype might be influencing the outcome. We have shown previously that culture conditions can influence GPEET expression [22]. When we monitored the expression of GPEET, we found that 3 cultures that were SoMo-positive were all GPEET-positive and conversely, 4 cultures that were SoMo-negative were all negative for GPEET.

We then systematically examined SoMo of early and late procyclic forms. For these experiments we derived early procyclic forms from bloodstream forms and let them differentiate into late procyclic forms by removal of glycerol. When these cultures were seeded onto plates containing glycerol, both early and late procyclic forms grew and formed colonies at the inoculation site, but only the former produced migrating fingers (Figure 2). It has previously been shown that glycerol alone does not trigger the reversion of late to early procyclic forms in liquid culture [4]. Nevertheless, to be sure that the status of the cells had not changed on the plates, a “community lift” was performed. This entails placing a nitrocellulose filter on the plate; when the filter is removed, the cells adhere to it and can be labelled with antibodies. Incubation of the filter with antibodies against GPEET and EP confirmed that the early procyclic forms were positive for both, as expected, and that most cells in the colony of late procyclic forms were negative for GPEET. Some GPEET-positive cells can always be detected in cultures without glycerol [7]; these are visible as a narrow ring at the edge of the colony in Figure 2. It is not clear if the few early procyclic forms actively migrate to the border of the colony or if cells at the edge are more likely to revert to expressing GPEET. Although late procyclic forms do not show SoMo, they are recognised by early procyclic forms, which react by changing their direction of migration (Figure 2).

### GPEET is not essential for social motility

**Figure 3:**
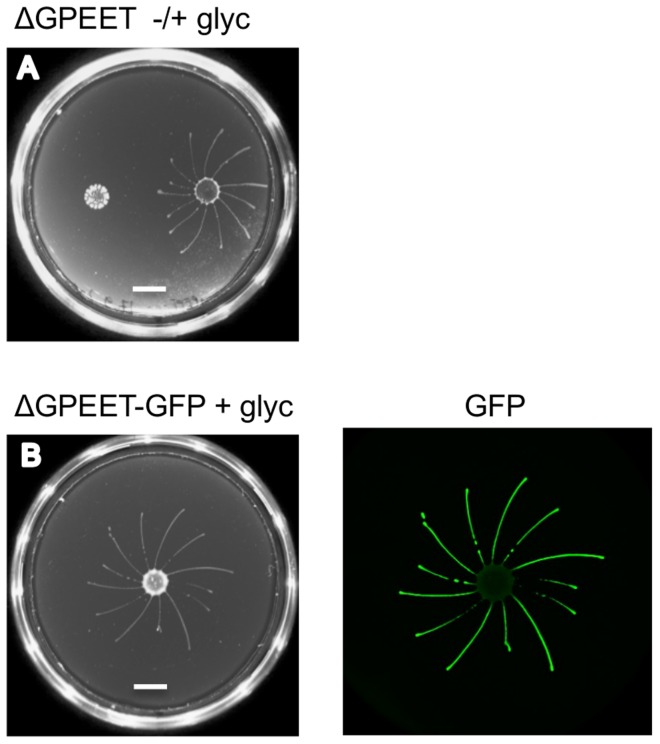
GPEET is not essential for SoMo. (A) 2×10^5^ ΔGPEET cells, cultured with or without glycerol, were inoculated onto a plate containing 20 mM glycerol. Four days post plating only the cells cultured in medium containing glycerol showed SoMo. The scale bar is 1 cm. (B) 4×10^5^ ΔGPEET/GFP cells, cultured in medium containing glycerol, were inoculated onto a plate containing 20 mM glycerol. Four days post plating a community lift was stained with α-GFP antibody.

GPEET is the major surface protein of early procyclic forms. To test if it was required for SoMo we used the ΔGPEET deletion mutant previously generated in our laboratory [6]. Since these cells lack a marker for early procyclic forms, we once again took bloodstream forms and triggered them to differentiate to procyclic forms. In common with its wild-type parent, ΔGPEET was SoMo-positive as long as it was cultured in the presence of glycerol and became SoMo-negative after being transferred to glycerol-free medium (Figure 3A). In order to track the differentiation status of ΔGPEET, it was transformed with a reporter construct in which the GFP coding region is fused to the GPEET 3′ untranslated region [23]. This regulatory sequence ensures that expression of GFP mimics that of GPEET, and indicates whether or not a cell is still an early procyclic form. A community lift using an anti-GFP antibody revealed once again that only the early procyclic forms migrate while the late, GFP-negative cells stay at the point of inoculation (Figure 3B). In addition to migrating, ΔGPEET cells are still capable of recognising and reacting to other trypanosome communities (Figure 3A).

### Other markers differentially expressed in early and late procyclic forms

**Figure 4:**
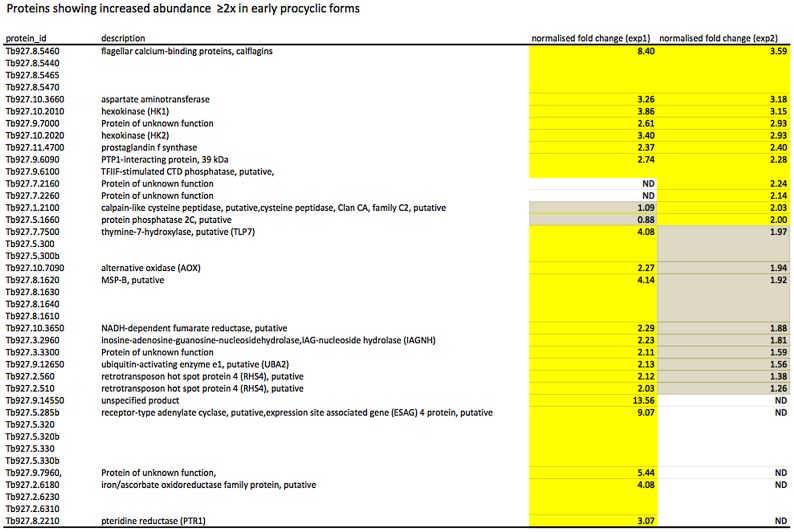
Proteins up-regulated in early procyclic forms. Yellow: proteins showing ≥2-fold increase in abundance. Grey: ≤2-fold change in abundance. N.D. not detected. Biological replicates (exp1 and exp2) were performed.

**Figure 5:**
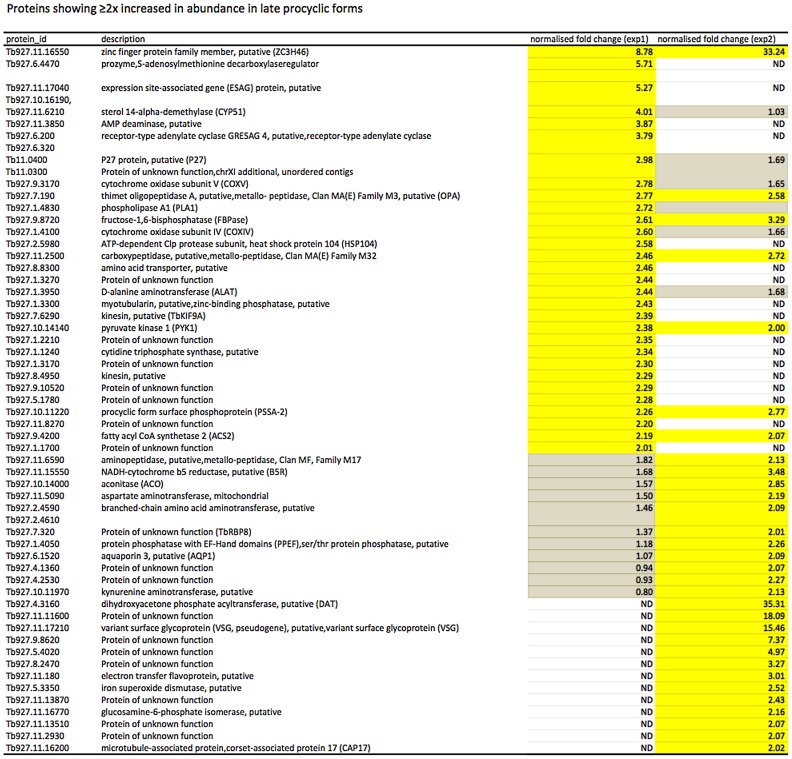
Proteins up-regulated in late procyclic forms. Yellow: proteins showing ≥2-fold increase in abundance. Grey: ≤2-fold change in abundance. N.D. not detected. Biological replicates (exp1 and exp2) were performed.

**Figure 6:**
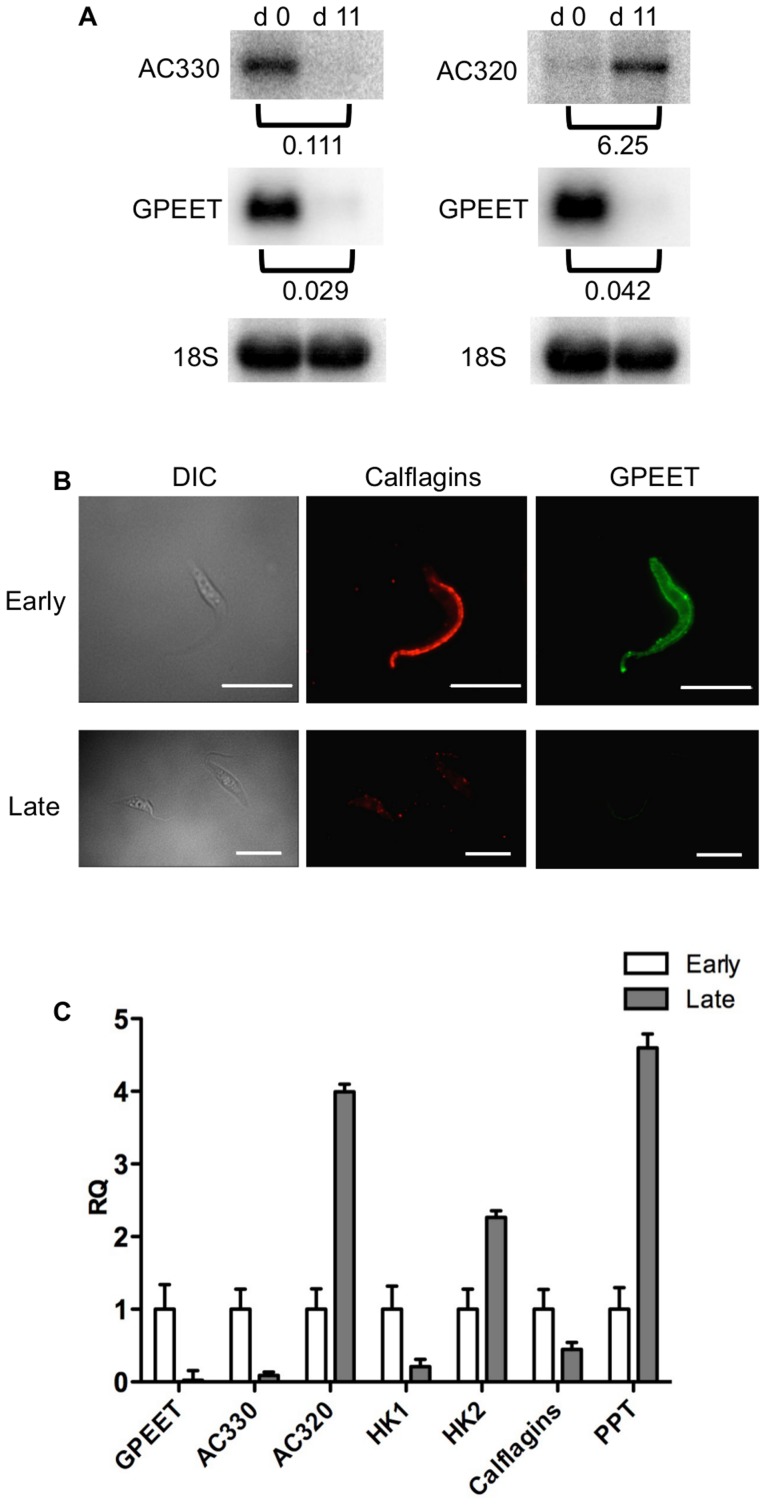
Differential expression of markers in early and late procyclic culture forms. (A) Northern blot analysis of adenylate cyclases Tb927.5.330 (AC 330) and Tb.927.5.320/285b (AC 320). GPEET was used to verify that the cultures correspond to early and late procyclic forms, respectively. Signals were quantified using a PhosphoImager and normalised against 18S rRNA as described previously [47]. Signals in early procyclic forms were set at 1. (B) Immunofluorescence analysis reveals that GPEET and calflagin are co-expressed by early procyclic culture forms and are not detectable in late procyclic culture forms. Scale bar: 10 µm. (C) Quantitative RT-PCR performed using RNA from early procyclic culture forms and late procyclic culture forms 11 days after removal of glycerol from the medium [4], [7]. RQ: Relative quantification. Expression levels in early procyclic forms are set at 1. α-tubulin was used to normalise mRNA levels. Error bars are ΔCt standard errors. AC 330: Tb927.5.330 3′ UTR; AC 320: Tb927.5.320/285b 3′ UTR; HK1: 3′ UTR of Tb927.10.2010: HK2: 3′ UTR of Tb927.10.2020; Calflagin: coding region of Tb927.8.5460, Tb927.8.5440, Tb927.8.5465, Tb927.8.5470. PPT: coding region of Tb927.1.2850, Tb927.1.2880 (putative pteridine transporters).

In culture, early and late procyclic forms are morphologically indistinguishable. Since GPEET was the only known marker for early procyclic forms at the beginning of this study, we used SILAC to identify additional proteins that were differentially expressed between GPEET-positive and GPEET-negative cells. Two independent experiments identified a limited number of candidates that were significantly different in at least one experiment (≥2-fold; Figures 4 and 5; Table S2). Of the differentially regulated proteins, three examples were encoded by related genes. These were the calflagins (Tb-44, Tb-24 and Tb-17), the two hexokinases (HK1 and HK2) and three adenylate cyclases. The members of a protein family could not be identified unequivocally as they contained identical peptides that are randomly assigned during mapping. Lacking antibodies that discriminated between isoforms, we analysed the transcripts for unique signatures. In the case of the adenylate cyclases (Tb927.5.285b, Tb927.5.320 and Tb 927.5.330 - here designated AC330, AC320 and AC285b) differences in their 3′ untranslated regions, allowed AC330 to be distinguished from AC320/285b by Northern blot analysis (Figure 6A). Both were differentially expressed, with AC330 up 9-fold in early procyclic forms and AC320/285b up 6.25-fold in late procyclic forms. Thus, the changes in protein levels detected by SILAC are probably an under-estimate for the individual proteins. Since an antiserum was available against calflagins, we monitored expression of these proteins by immunofluorescence. This revealed that calflagin-positive cells were always also positive for GPEET (Figure 6B). In addition, we performed quantitative RT-PCR to measure transcript levels in early and late procyclic forms (Figure 6C). This confirmed the differential expression at the level of mRNA for the adenylate cyclases and HK1/HK2. HK1 mRNA was expressed 4-fold more in early procyclic forms while HK2 was up-regulated 2-fold in late procyclic forms. In contrast to what was observed by immunofluorescence and SILAC, calflagin transcripts were down-regulated only 2-fold in late procyclic forms, suggesting that there is an additional level of regulation. Finally, we tested the mRNA levels of a set of putative pteridine transporters (PPT: Tb927.1.2850, Tb927.1.2880), which we have observed to be up-regulated (at least transiently) during differentiation of early to late procyclic forms; these were increased 4.6-fold in late procyclic forms. In summary, although no other gene is as tightly regulated as GPEET, we have identified several additional differentially regulated transcripts/proteins in early and late procyclic forms.

**Figure 7:**
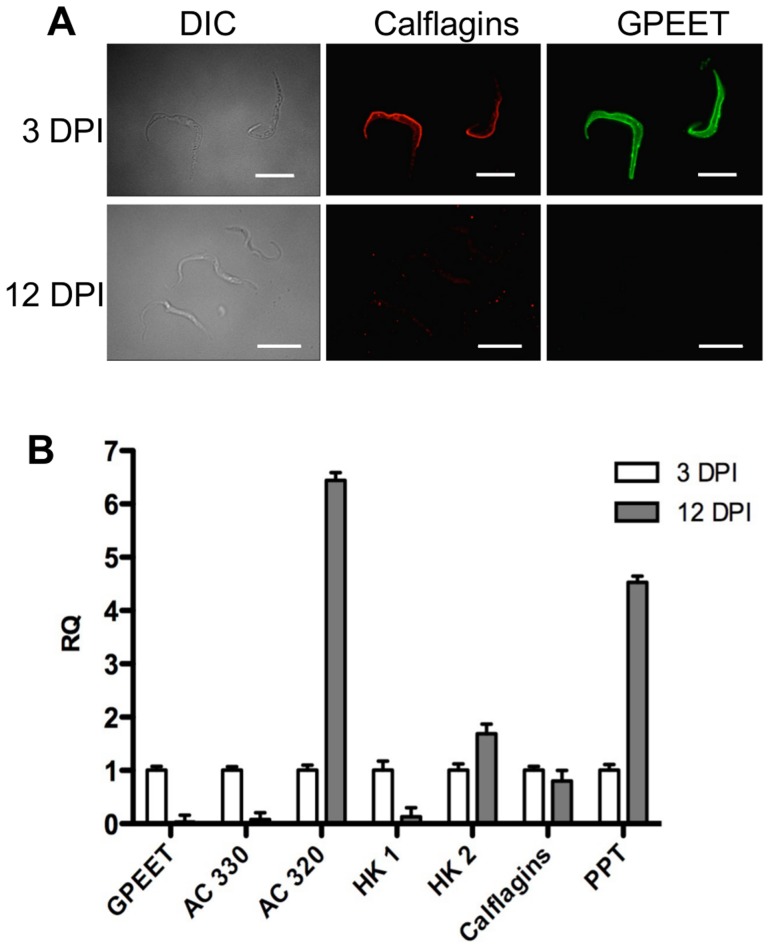
Differential expression of early and late procyclic form markers in tsetse flies. A. GPEET and calflagin are co-expressed by early procyclic forms 3 days post infection (DPI), but neither is detectable in late procyclic forms 12 DPI. Trypanosomes were isolated from tsetse fly midguts, fixed with formaldehyde and glutaraldehyde and permeabilised with Triton-×100. Immunofluorescence was performed with anti-GPEET and anti-calflagin antisera. Scale bar: 10 µm. B. Quantitative RT-PCR was performed with RNA isolated from infected tsetse flies 3 and 12 days post infection. Gene designations are the same as for Figure 6. RQ: relative quantification. α-tubulin was used to normalise mRNA levels. Error bars are ΔCt standard errors.

It has been shown previously that expression of the GPEET transcript and protein in the fly mirrors that of cells differentiating from early to late procyclic forms in culture [4], [22], [24]. To test if the new markers that we identified were similarly regulated in vivo, tsetse flies were infected and trypanosomes were harvested 3 and 12 days post infection. Figure 7A shows the co-expression of GPEET and calflagin in early procyclic forms isolated from fly midguts at day 3 and the repression of both proteins by day 12. Quantitative RT-PCR (Figure 7B) showed the same profiles that were observed in culture, with GPEET, AC330 and HK1 being more highly expressed in early procyclic forms and AC320, HK2 and PPT being more highly expressed in late procyclic forms. Taken together, these data convincingly show that early procyclic forms in culture are equivalent to the procyclic forms early in infection and late procyclic forms correspond to those in established infections.

### Mutants with a defect in salivary gland infection are SoMo-positive

**Figure 8:**
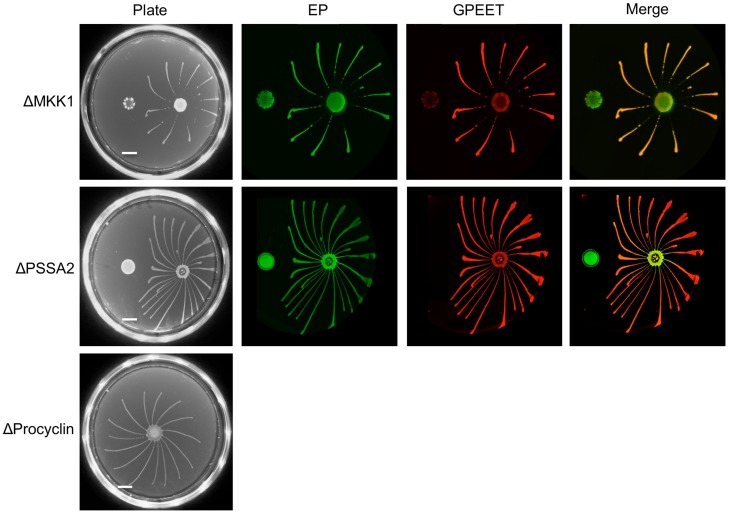
Deletion mutants with defects in salivary gland infection rates are SoMo-positive. A and B. MKK1 and PSSA-2 deletion mutants were cultured with or without glycerol. 4×10^5^ cells from each culture were inoculated onto plates containing 20 mM glycerol. The scale bar is 1 cm. Four days post plating community lifts were incubated with α-EP and α-GPEET antibodies. C. 2×10^5^ cells of the procyclin null mutant (Δproc), obtained by differentiation of bloodstream forms, were inoculated onto a 0.4% agarose plate containing 20 mM glycerol. A photograph was taken 4 days post plating.

Despite the lack of SoMo by late procyclic forms, it is possible that it plays a role in migration of proventricular forms across the cardia to the tsetse salivary glands. To test this hypothesis we used a series of deletion mutants with defects in salivary gland infection. Our previous studies have implicated at least two proteins in the establishment of mature salivary gland infections, mitogen-activated kinase kinase 1 (MKK1; [25]) and the surface protein PSSA-2 [26]. Parasites lacking MKK1 were completely unable to establish salivary gland infections and parasites lacking PSSA-2 showed reductions in the prevalence and intensity of infections. A procyclin null mutant, lacking all EP and GPEET genes (Δproc), also showed a defect in colonisation of the salivary glands [6]. ΔMKK1 and ΔPSSA-2 infect the midgut at normal rates and intensities [25], [26], while Δproc establishes heavy infections at about half the rate of its wild-type parent [6]. MKK1 AND PSSA-2 knockouts were plated as early and late procyclic forms; in the case of Δproc only early procyclic forms, derived directly from bloodstream forms, were tested (Figure 8). In all cases, the early procyclic forms were positive for SoMo and were also able to sense and avoid the communities of late procyclic forms on the same plate.

## Discussion

Early procyclic forms - defined as GPEET-positive cells - are detected in the midgut of tsetse flies in the first week after uptake of bloodstream form trypanosomes [4], while establishment of a persistent infection correlates with differentiation to late (GPEET-negative) procyclic forms. We have discovered that SoMo is a property of early procyclic forms and that late procyclic forms are consistently SoMo-negative. There are several indications that SoMo reflects the migration of trypanosomes from the midgut lumen to the ectoperitrophic space in the first days of fly infection rather than the subsequent migration from the ectoperitrophic space to the salivary glands. First and foremost, SoMo is restricted to early procyclic forms whereas late procyclic forms, which are forerunners of the forms migrating to the salivary glands, are SoMo-negative. Second, the timing of the switch from early to late procyclic forms [4] correlates with the appearance of parasites in the ectoperitrophic space [5]. Third, SoMo is independent of GPEET, as is colonisation of the ectoperitrophic space [4]. Furthermore, three mutants (Δproc, ΔPSSA-2 and ΔMKK1) that show normal colonisation of the midgut, but defects in colonisation of the salivary glands [6], [25], [26] are SoMo-positive as early procyclic forms. While it might be argued that these mutants have other defects, such as an inability to penetrate the proventriculus or to differentiate to epimastigote forms, in no case does the mutation impair SoMo by early procyclic forms.

Social interactions between bacteria are known to involve outer membrane proteins [27]. Despite being the major surface glycoproteins of procyclic forms, and present in several million copies, neither GPEET nor EP procyclin is required by trypanosomes for SoMo. It is known, however, that procyclin null mutants export free GPI anchors to their surface [28], and these might compensate for the loss of procyclins. The insect-stage specific transmembrane protein PSSA-2 [26], which shows increased expression in late procyclic forms (Table 2), is also dispensable for SoMo.

In this study we identified additional proteins and transcripts that are differentially expressed in these two life-cycle stages, both in culture and in the fly. Like GPEET, calflagins are expressed by early procyclic forms, but are down-regulated in late procyclic forms. It was recently shown by Emmer and coworkers that calflagins are expressed by bloodstream and procyclic forms [29]. However, when Kolev et al. induced differentiation from procyclic to epimastigote and metacyclic forms by overexpression of RBP6 [30], the procyclic forms in their cultures were heterogeneous with respect to calflagin expression, suggesting that they were a mixed population of early and late forms. Calflagins were not detected in epimastigotes but were re-expressed by metacyclic forms in culture [30] and in the fly salivary glands [31]. Guided by SILAC, we also identified two pairs of proteins, HK1/HK2 and AC330/AC320, whose transcripts are reciprocally expressed in early and late procyclic forms. This is similar to the situation that is seen with GPEET and EP3 [4], [24], [32]. Given that we only detected about 1300 proteins by mass spectrometry in the two experiments (Table S2), we do not claim that the list of differentially regulated proteins is complete, and indeed we suspect that there might be other sets of proteins that are reciprocally regulated without a discernible net change. Moreover, there might also be post-translational modifications or non-peptide moieties that are stage-specific. For example, the activity of the kinase that phosphorylates GPEET is restricted to early procyclic forms [33]. It is also known that early procyclic forms of *T. congolense* preferentially express PRS, a protease-resistant surface glycoconjugate [34], [35], although an equivalent molecule has not been reported for *T. brucei*.

Our experiments show that the parasites need to reach a threshold concentration on plates before they start to migrate. Although the number of parasites in the midgut lumen is significantly lower during the early days of infection [5], [36], the three-dimensional structure of the midgut and host-derived factors might contribute to the response. Moreover, local accumulation of parasites, for example at the peritrophic matrix, could condition the micro-environment in a manner conducive to SoMo. In addition to migrating, early procyclic forms have the capacity to recognise and be repelled by communities of early or late procyclic forms. At present we can only speculate about the significance of such repellents, but one possibility is that they are used by late procyclic forms to prevent or reduce superinfection by a second strain of trypanosomes. Mixed infections of tsetse can be detected in the field [37], [38], but there is no information on whether flies acquire the parasites simultaneously or sequentially from infected mammals.

Although the differentiation of early to late procyclic forms in the tsetse fly is irreversible, it should always be borne in mind that trypanosomes can switch between these two life-cycle stages in liquid culture [22]. The fact that these can change over time has important implications for the interpretation of results. In particular, before it can be concluded that a specific gene is required for SoMo, it is essential to determine whether the parasites are early or late procyclic forms.

In conclusion, our findings add further credence to the designation of early and late procyclic forms as two distinct life cycle stages with different biological properties. Since early procyclic forms are only detected in the first week of tsetse fly infection [4], [32], this strongly suggests that SoMo reflects an early event in the colonisation of the insect host. It also implies that genes that are important for SoMo will also play a role in the establishment of a midgut infection. Using the SoMo assay as a surrogate for fly experiments would enable many more laboratories to examine this aspect of parasite transmission. In addition, related parasites such as the South American trypanosomes and Leishmania, which are also transmitted by insects, may be amenable to such studies.

## Materials and Methods

### Ethics statement

No vertebrate animals were used in this study. Bloodstream form trypanosomes were obtained from frozen stabilates stored in liquid nitrogen. Antibodies were obtained from cell culture supernatants or from pre-existing sources of serum.

### Trypanosomes

The pleomorphic strain AnTat 1.1 [39], [40] and genetically manipulated derivatives of it were used in this study. The deletion mutants ΔPSSA-2 [26], ΔMKK1 [25], ΔGPEET and Δproc [6] have all been described previously. Procyclic forms were cultured in SDM79 [41] supplemented with 10% heat inactivated foetal bovine serum (iFBS). The medium for early procyclic forms was also supplemented with 20 mM glycerol [4].

### Infection of tsetse flies and RNA isolation

Pupae of *Glossina morsitans morsitans* were obtained from the Department of Entomology, Slovak Academy of Science (Bratislava). Teneral flies were infected with early procyclic forms during the first blood meal as described [42]. Flies were dissected and total RNA was isolated from midguts using standard procedures [43]. Fifty midguts were collected 3 days post infection for RNA isolation and immunofluorescence analysis. Approximately 10–15 infected midguts were collected for analysis at days 12 or 13 post infection.

### Differentiation

Bloodstream forms obtained from frozen stabilates (500 µl blood plus 500 µl HMI-9) were centrifuged and resuspended in SDM79 supplemented with 10% iFBS and 20 mM glycerol. Differentiation to early procyclic forms was induced by the addition of 6 mM *cis*-aconitate and a temperature shift from 37° to 27° [44]. Early procyclic forms were cultured in the same medium [4]. To trigger differentiation to late procyclic forms, early procyclic forms were transferred to SDM79, 10% iFBS without glycerol, as described previously [4].

### Plasmids and generation of stable transformants

Stable transformation of procyclic form trypanosomes was performed as described [26]. To generate the ΔGPEET/GFP-GPEET cell line, which expresses GFP under the control of the GPEET promoter and 3′ UTR, ΔGPEET [6] was stably transformed with the plasmid pCorleone-GFP/GPEET-blast [23]. When linearised with Spe I, this plasmid integrates upstream of a procyclin locus.

### Plates

The protocol to produce plates was adapted from [20]. Plates were always used within 24 h. Briefly, 36 ml SDM79 supplemented with 10% iFBS were pre-warmed to 42°; for plates containing glycerol, 400 µl of a 2M glycerol stock was added. 4 ml agarose (Promega V3125; 4% w/v in water) was added to the pre-warmed medium and the resulting 0.4% agarose medium was immediately poured into Petri dishes with a diameter of 85 mm, 10 ml per dish. The open plates were then air-dried for 1 hour in a laminar flow cabinet. Cells from an exponentially growing culture were centrifuged briefly and resuspended in the residual medium at a density of 3–4×10^7^ cells ml^−1^. Five µl were spotted onto the surface of the agarose, the Petri dish was sealed with Parafilm and incubated at 27°. All experiments were performed at least twice and there were no incongruent results. It should be noted, however, that the number of spokes produced by a given clone can vary between experiments.

### Community lifts

A nitrocellulose filter (Whatman Protran BA 85) was laid carefully on top of the cells on the agarose plate and incubated for 5 minutes at room temperature. The filter was then removed and air-dried for 15 minutes. The membrane was blocked in PBS containing 5% (w/v) defatted milk for 1 h at 4°, after which the primary antibodies were added at the appropriate dilution and incubated for 1 h at room temperature. The following primary antibodies were used: TBRP1/247 mouse α-EP 1∶500 [45], K1 rabbit α-GPEET 1∶1000 [42] and mouse α-GFP (Roche, 1∶2000). After incubation with the primary antibodies the membrane was washed 3 times in TBS Tween, then incubated with secondary antibodies (in PBS 5% milk) for 1 h at room temperature. The following secondary antibodies were used at a dilution of 1∶10000: goat α-mouse IRDye 800CW (LI-COR Biosciences) and goat α-rabbit IRDye 680LT (LI-COR Biosciences). The membrane was washed 3 times in TBS Tween and then scanned on a LI-COR Odyssey Infrared Imager model 9120, using Odyssey Application Software, Version 3.0.21.

### Imaging and image processing

Images from plates were made with a Nikon MH-56 digital camera. To quantify the intensity of the community lifts a grey scale image of the membranes was exported from the Odyssey Application Software and analysed with ImageJ 1.46r. Seven individual areas were analysed for each value. The values were subtracted from 255 to obtain a maximum intensity of 255 and a minimum intensity of 1. The graphs were generated with Prism6.

### Stable isotope labeling by amino acids in culture (SILAC) and mass spectrometry

Late procyclic forms were derived from early procyclic forms by removal of glycerol in two independent experiments. Pairs of early and late procyclic forms were adapted to SDM80 supplemented with 10% dialysed foetal bovine serum. The medium for early procyclic forms was supplemented with 20 mM glycerol. SILAC and mass spectrometry analyses were performed as described [46] at the Mass Spectrometry and Proteomics Facility, Department of Clinical Research, University of Bern.

### Northern blot analysis and qRT-PCR

The isolation of RNA from early and late procyclic culture forms and northern blot analysis were performed as described [6]. Purified RNA was subjected to DNAse treatment prior to cDNA synthesis. Reverse transcription was performed using an Omniscript RT kit (Quiagen, Switzerland) according to the manufacturer's instructions with random hexamers as primers. PCR primers are shown in Table S1. qPCR was performed using MESA GREEN qPCR MasterMix Plus for SYBR Assay (Eurogentec) in the ABI Prism 7000 Sequence Detection System (Applied Biosystems). Specificity of the reactions was confirmed by agarose gel electrophoresis and melting temperature analysis. The data were analysed using 7000 System SDS software v1.2 (Applied Biosytems). Two biological replicates were analysed independently. Within an experiment, technical triplicates were run in parallel.

### Immunofluorescence

Cells were washed twice with PBS and spread on a coverslip to let them settle down for 10 minutes. The cells were fixed with 4% paraformaldehyde and 0.1% glutaraldehyde in PBS for 15 minutes, then permeabilised with 0.2% Triton X-100 and blocked with 2% BSA/PBS. The primary antiserum, rabbit K1 anti-GPEET was diluted 1∶1000 [42] and the calflagin mouse antiserum (a gift from David Engman), was diluted 1∶500 [29]. The secondary antibodies Alexa Fluor 488 goat anti-rabbit and Cy3 goat anti-mouse (Invitrogen) were diluted 1∶1000 in 2% BSA/PBS. Images were taken with a Leica DFC360FX monochrome CCD (charge-coupled-device) camera mounted on a Leica DM5500 B microscope with a 100× oil immersion objective and analysed using LAS AF software (Leica).

## Supporting Information

Table S1 List of primers used for quantitative RT-PCR.
(XLSX)

Table S2 Proteins detected by SILAC.
(XLSX)
